# Genome sequences of four novel *Endozoicomonas* strains associated with a tropical octocoral in a long-term aquarium facility

**DOI:** 10.1128/mra.00833-24

**Published:** 2024-12-09

**Authors:** Matilde Marques, Daniela M.G. da Silva, Elsa Santos, Núria Baylina, Raquel Peixoto, Nikos C. Kyrpides, Tanja Woyke, William B. Whitman, Tina Keller-Costa, Rodrigo Costa

**Affiliations:** 1 Institute for Bioengineering and Biosciences and i4HB-Institute for Health and Bioeconomy, Instituto Superior Técnico, Universidade de Lisboa, Lisboa, Portugal; 2 Department of Bioengineering, Instituto Superior Técnico, Universidade de Lisboa, Lisboa, Portugal; 3 Oceanário de Lisboa, Esplanada D. Carlos I, Lisbon, Portugal; 4 King Abdullah University of Science and Technology, Red Sea Research Center, Thuwal, Saudi Arabia; 5 Department of Energy Joint Genome Institute, Lawrence Berkeley National Laboratory, Berkeley, California, USA; 6 Department of Microbiology, University of Georgia, Athens, Georgia, USA; California State University San Marcos, San Marcos, California, USA

**Keywords:** Chitinases, *Endozoicomonadaceae*, host-microbe interactions, coral holobiont, symbiosis, bacteria

## Abstract

We report the genome sequences of four *Endozoicomonas* sp. strains isolated from the octocoral *Litophyton* maintained long term at an aquarium facility. Our analysis reveals the coding potential for versatile polysaccharide metabolism; Type II, III, IV, and VI secretion systems; and the biosynthesis of novel ribosomally synthesized and post-translationally modified peptides.

## ANNOUNCEMENT

The bacterial genus *Endozoicomonas* (*Pseudomonadota*, *Endozoicomonadaceae*) is a subject of increasing research interest owing to its widespread association with marine animals, particularly corals (1–4). However, *Endozoicomonas* spp. are typically difficult to cultivate and maintain in the laboratory (3, 4).

We report the genomes of four *Endozoicomonas* strains isolated from two *Litophyton* sp. specimens kept in a 19-m^3^ tropical exhibition aquarium at the Oceanário de Lisboa, Portugal. Host-derived microbial cell suspensions were retrieved as described previously (2). One gram of coral tissue was homogenized in 9 mL of sterile Ca^2+^- and Mg^2+^-free artificial seawater (2). The homogenate was serially diluted, plated separately on marine agar diluted 1:2 and R2A diluted 1:10 media, and incubated at 21°C for 4 weeks. Genomic DNA of single colonies was extracted from cultures freshly grown in 1:2 marine broth using the Wizard Genomic DNA Purification kit (Promega, USA). Purity was confirmed by Sanger sequencing of 16S rRNA genes amplified from genomic DNA using universal primers (F27 and R1492). Taxonomy assignment was performed with the SILVA Alignment, Classification, and Tree Service (v1.2.12) and database (v138.1). The same genomic DNA samples were used for genome sequencing at the DOE Joint Genome Institute (JGI) using PacBio sequencing technology (5). For each sample, genomic DNA was sheared to 6–10 kb, treated using SMRTbell Express Template Prep Kit 3.0, and purified with SMRTbell cleanup beads (PacBio). The purified product was enriched using barcoded amplification oligos (IDT) and SMRTbell gDNA Sample Amplification Kit (PacBio). A 10-kb PacBio SMRTbell library was constructed and sequenced on the PacBio Revio system using HiFi chemistry. Raw reads were quality-filtered as per the JGI standard operating practice (SOP) protocol 1061 using BBTools v.38.86 (http://bbtools.jgi.doe.gov). Filtered reads >5 kb were assembled using Flye v2.8.3 (6). Organism and project metadata were deposited in the Genomes OnLine database (7). Contigs were annotated using the NCBI Prokaryotic Genome Annotation Pipeline (PGAP v.6.7) (8) and the DOE-JGI Microbial Genome Annotation Pipeline (MGAP v.4) (9) coupled to the Integrated Microbial Genomes and Microbiomes system v7 (IMG/M) for comparative analyses (10). Genome completeness and contamination were assessed with CheckM (v1.2.3) (11). AntiSMASH v7.1 (12) was used to identify secondary metabolite biosynthetic gene clusters (SM-BGCs). Default parameters were used for all software, unless otherwise specified.

Sequencing statistics and genome features are shown in Table 1. Average nucleotide identities (ANIs), calculated with FastANI v0.1.3 on KBase (13, 14), among strains NE35, NE40, NE41, and NE43, were above 99.9% in all pairwise comparisons. All four strains shared approximately 89.3% ANI with their closest relative, as determined by phylogenomics, including all *Endozoicomonas-*type strains with a publicly available genome: *Endozoicomonas gorgoniicola* PS125^T^ (GCA_025562715), also isolated from an octocoral (15).

**TABLE 1:** General sequencing statistics and genome features of the *Endozoicomonas* sp. reported in this study

Strain^*a*^	Genome size (Mb)	GC content (%)	Genome coverage (x)	Number of contigs	Contig N50 (Mb)	Number of reads^*b*^	Average read length (bp)^*b*^	Estimated Completeness (%)	Estimated Contamination (%)	Counts^*c*^								GenBank accession number	SRA accession number	Bioproject accession number	Biosample accession number
										Genes	CDs	RNA	rRNA	tRNA	ncRNA	COG^*d*^	Pfam^*d*^				
**NE35**	5.5	49.0	187.0	4	5	1,788,957| 3,242	10,318 ± 3,193.2| 9,971 ± 3,275.8	99.08	4.41	4,955*| 4,861†	4,828*| 4,667†	137†	25*| 25†	97*| 107†	5†	3,458٭	4,933٭	JBEWTA000000000	SRR28058472 SRR28058473	PRJNA1075803	SAMN39945177
**NE40**	5.5	49.0	202.0	3	5.1	7,826,899| 10,098	9,477 ± 2,410.7| 9,290 ± 2,556.6	99.14	4.19	4,947*| 4,849†	4,820*| 4,657†	137†	25*| 25†	97*| 107†	5†	3,458٭	4,933٭	JBEWTB000000000	SRR28058719 SRR28058720	PRJNA1075804	SAMN39945184
**NE41**	5.5	49.0	195.0	6	5	3,594,929| 5,045	10,617 ± 2,883.3| 10,003 ± 2,860.4	99.03	4.08	4,981*| 4,888†	4,856*| 4,699†	137†	25*| 25†	97*| 107†	5†	3,449٭	4,929٭	JBEWTC000000000	SRR28058712 SRR28058713	PRJNA1075805	SAMN39945181
**NE43**	5.5	49.0	196.0	3	5.1	4,034,152| 6,017	10,290 ± 2,785.9| 9,884 ± 2,807.1	99.21	4.41	4,941*| 4,855†	4,814*| 4,667†	137†	25*| 25†	122*| 107†	5†	3,463٭	4,939٭	JBEWTD000000000	SRR28058717 SRR28058718	PRJNA1075806	SAMN39945185

^*a*^ All strains reported in this study have been isolated from the octocoral host *Litophyton* sp. Strains NE35, NE41, and NE43 were isolated from the same specimen on MA 1:2, whereas strain NE40 was isolated from a second specimen on R2A 1:10 medium.

^*b*^ Values per run on two different SMRT cells. SRA accessions are provided per run.

^*c*^ Annotation was performed using the DOE-JGI Microbial Genome Annotation Pipeline (MGAP v.4) (٭) and the NCBI Prokaryotic Genome Annotation Pipeline (PGAP v.6.7) (†).

^*d*^ Annotation files are publicly accessible on Zenodo (https://doi.org/10.5281/zenodo.13863125).

**Fig 1:**
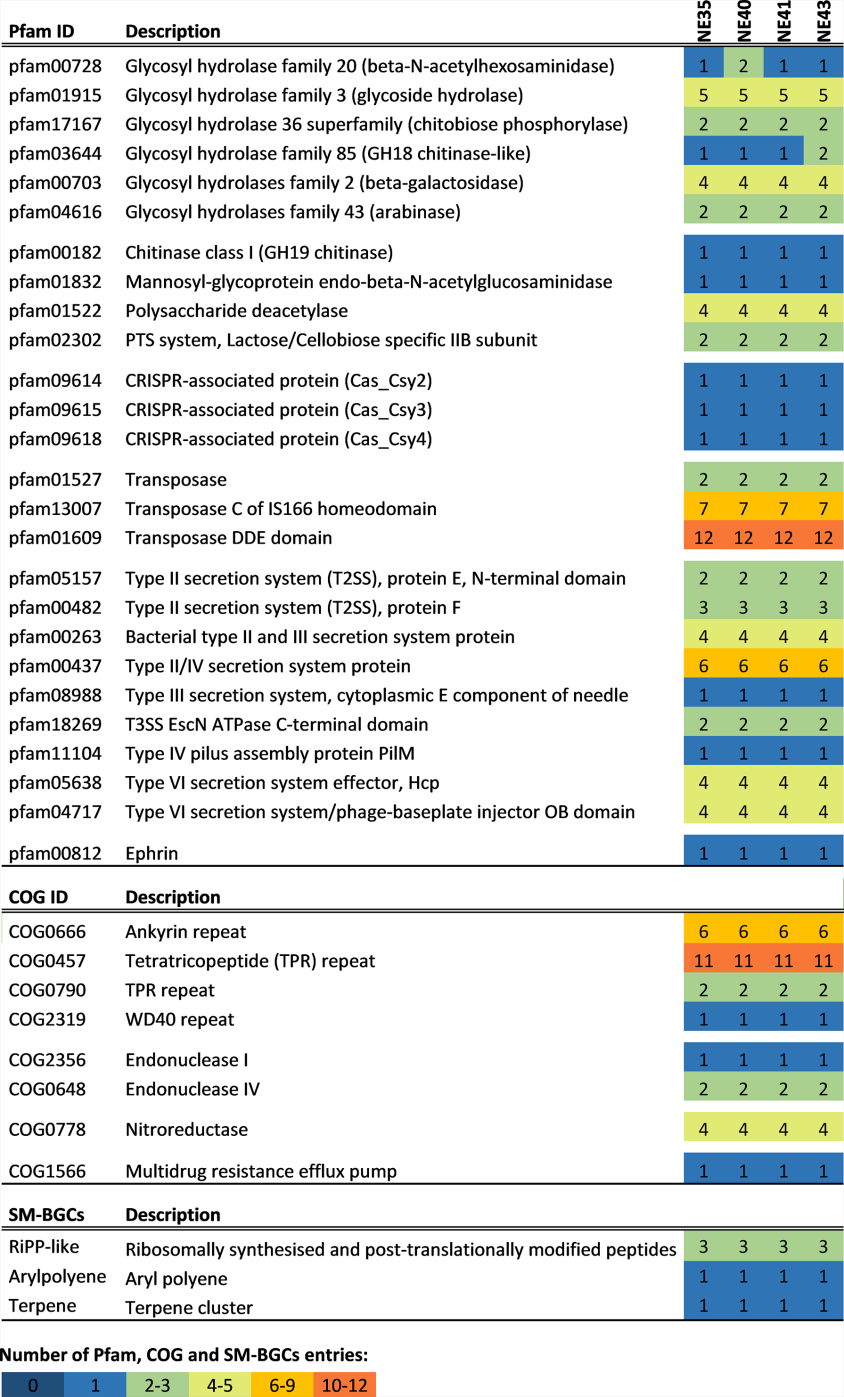
Presence and abundance of select functional features of the *Endozoicomonas* sp. genomes described in this study. Values of each entry represent the numbers of coding sequences assigned to Pfam (top) and COG (middle) functions per genome (https://doi.org/10.5281/zenodo.13863125), and the number of SM-BGCs (bottom) coding for major compound classes identified with antiSMASH v.7.0 (https://doi.org/10.5281/zenodo.13683288).

All four genomes encode several glycoside hydrolases, featuring chitinase, polysaccharide deacetylase, N-acetylglucosaminidase, and beta-galactosidase-encoding genes, congruent with the emerging view of complex carbon metabolism among *Endozoicomonadaceae* spp. associated with marine invertebrates (16–18). Multiple protein domains underlying Type II, III, IV, and VI secretion systems were predicted to be encoded in all genomes. Additionally, three CRISPR–Cas antiviral defense systems, several eukaryotic-like repeat protein motifs, and the potential to synthesize putatively novel ribosomally synthesized and post-translationally modified peptides, among other natural products, were encoded (Fig. 1).

## Data Availability

The genome sequences of the four *Endozoicomonas* sp. strains have been deposited in GenBank/NCBI. GenBank accession numbers are listed in Table 1. The assemblies of NE35, NE40, NE41, and NE43 are available under the BioProject accession numbers PRJNA1075803, PRJNA1075804, PRJNA1075805, and PRJNA1075806, respectively. The raw reads are available under accession numbers SRR28058472 and SRR28058473 for NE35, SRR28058719 and SRR28058720 for NE40, SRR28058712 and SRR28058713 for NE41, and under SRR28058717 and SRR28058718 for NE43. COG and Pfam annotation results on IMG/M v7 and AntiSMASH results are available under https://doi.org/10.5281/zenodo.13863125 and https://doi.org/10.5281/zenodo.13683288, respectively.
